# GAD2 Expression Defines a Class of Excitatory Lateral Habenula Neurons in Mice that Project to the Raphe and Pontine Tegmentum

**DOI:** 10.1523/ENEURO.0527-19.2020

**Published:** 2020-05-20

**Authors:** Lely A. Quina, Andrew Walker, Glenn Morton, Victor Han, Eric. E. Turner

**Affiliations:** 1 Center for Integrative Brain Research, Seattle Children’s Research Institute, Seattle, WA 98101; 2 Department of Psychiatry and Behavioral Sciences, University of Washington, Seattle, WA 98195

**Keywords:** GABA, glutamate, lateral habenula, nucleus incertus, raphe

## Abstract

The lateral habenula (LHb) sends complex projections to several areas of the mesopontine tegmentum, the raphe, and the hypothalamus. However, few markers have been available to distinguish subsets of LHb neurons that may serve these pathways. In order to address this complexity, we examined the mouse and rat LHb for neurons that express the GABA biosynthesis enzymes glutamate decarboxylase 1 (GAD1) and GAD2, and the vesicular GABA transporter (VGAT). The mouse LHb contains a population of neurons that express GAD2, while the rat LHb contains discrete populations of neurons that express GAD1 and VGAT. However, we could not detect single neurons in either species that co-express a GABA synthetic enzyme and VGAT, suggesting that these LHb neurons do not use GABA for conventional synaptic transmission. Instead, all of the neuronal types expressing a GABAergic marker in both species showed co-expression of the glutamate transporter VGluT2. Anterograde tract-tracing of the projections of GAD2-expressing LHb neurons in *Gad2^Cre^* mice, combined with retrograde tracing from selected downstream nuclei, show that LHb-GAD2 neurons project selectively to the midline structures in the mesopontine tegmentum, including the median raphe (MnR) and nucleus incertus (NI), and only sparsely innervate the hypothalamus, rostromedial tegmental nucleus (RMTg), and ventral tegmental area (VTA). Postsynaptic recording of LHb-GAD2 neuronal input to tegmental neurons confirms that glutamate, not GABA, is the fast neurotransmitter in this circuit. Thus, GAD2 expression can serve as a marker for functional studies of excitatory neurons serving specific LHb output pathways in mice.

## Significance Statement

The lateral habenula (LHb) provides a major link between subcortical forebrain areas and the dopamine (DA) and serotonin (5HT) systems of the midbrain and pons, and it has been implicated in reward mechanisms and the regulation of mood states. Few markers have been available for the specific cell types and complex output pathways of the LHb. Here, we examined the expression of genes mediating GABAergic and glutamatergic transmission in the mouse and rat LHb, where no neurons in either species expressed a full complement of GABAergic markers, and all expressed the glutamatergic marker VGluT2. Consistent with this, in mice the LHb glutamate decarboxylase 2 (GAD2) neurons are excitatory and appear to use only glutamate for fast synaptic transmission.

## Introduction

The lateral habenula (LHb) is a diencephalic nucleus that provides a major link between the limbic forebrain and the dopamine (DA) and serotonin (5HT) systems of the ventral midbrain (Lecourtier and Kelly, 2007; Hikosaka et al., 2008). Most studies of LHb function have focused on the efferent pathway from the LHb to the ventral tegmental area (VTA), and particularly the rostromedial tegmental nucleus (RMTg), also known as the “GABAergic tail” of the VTA. LHb activity has long been known to inhibit the VTA (Christoph et al., 1986). Because LHb neurons projecting to the VTA are excitatory (Geisler et al., 2007), GABAergic interneurons, mainly located in the RMTg, are required to convert the LHb signal to an inhibitory one that can attenuate the firing of DA neurons (Perrotti et al., 2005; Jhou et al., 2009a,b; Li et al., 2019a). Through this intermediary, the LHb generates signals with a negative value (Proulx et al., 2014), which may include reward prediction error and/or punishment in both rodents (Tian and Uchida, 2015) and primates (Matsumoto and Hikosaka, 2007, 2009).

Anatomical studies of the projections of the LHb in rodents show that the well-studied RMTg DA modulation pathway is only a part of the complex LHb efferent system. Classic tracing studies in the rat have also identified caudal projections to the dorsal tegmental area (DTg), and the median raphe (MnR) and dorsal raphe (DR), plus rostral projections to the hypothalamus (Herkenham and Nauta, 1979). More recent detailed retrograde mapping of LHb projections in the rat (Bernard and Veh, 2012; Gonçalves et al., 2012) and the mouse (Quina et al., 2015) show that only a minority of LHb neurons project to the VTA/RMTg, and that the RMTg-projecting neurons are found predominantly in the lateral subnucleus of the LHb (LHbL), while neurons projecting to the more extensive raphe and DTg target areas tend to be distributed medially (LHbM). This extensive projection system implies that the LHb interacts with 5HT and other brain systems as well as DA (Metzger et al., 2017).

Experiments to determine the function of these diverse LHb pathways have been hindered by the lack of specific markers for manipulating the subpopulations of LHb neurons that comprise them. Microarray analysis of the habenula, and bioinformatic analysis of the Allen gene expression database for habenula-enriched transcripts, have revealed only a few such candidates, and many of these are shared with the medial habenula (Quina et al., 2009). The LHb is generally recognized to contain glutamatergic neurons. However, the search for functional heterogeneity has led to the identification of small populations of cells expressing GABAergic markers in the rat LHb, of uncertain significance (Zahm and Root, 2017). GABAergic neurons in most parts of the brain are characterized by the co-expression of two GABA biosynthetic enzymes, glutamate decarboxylase 1 (GAD1) and GAD2, and the vesicular GABA transporter (VGAT; Slc32a1). GAD1-expressing (Brinschwitz et al., 2010) and VGAT-expressing cells (Zhang et al., 2018) have been identified in the rat LHb, but it is unclear whether these putative LHb GABAergic neurons co-express all three GABAergic markers, nor is it known if these markers characterize comparable neurons in the mouse brain.

Here, we have systematically examined the expression and co-expression of GABAergic markers in the mouse and rat LHb. The mouse LHb contains a population of GAD2-expressing neurons, concentrated in the rostral and medial part of the nucleus, but these neurons do not express detectable levels of GAD1 or VGAT. Surprisingly, this pattern of GAD2 expression is absent in the rat LHb, where GAD2 is not detectable but GAD1 and VGAT are each expressed in discrete LHb neuronal populations. No neurons could be detected in either species that co-expressed VGAT together with a GABA synthetic enzyme. Instead, all of the neurons expressing a GABAergic marker also express the vesicular glutamate transporter VGluT2. Electrophysiological recording of the synaptic input from mouse LHb GAD2-expressing neurons to recipient neurons in the tegmentum confirms that glutamate is the sole fast neurotransmitter used by these cells, without evidence for synaptic release of GABA. Anterograde and retrograde tracing experiments using mice expressing *Gad2^Cre^* show that the LHb GAD2 neurons project predominantly to the raphe and dorsal tegmentum, including the nucleus incertus (NI), less so to the hypothalamus, and only sparsely if at all to the VTA/RMTg. Thus, GAD2 may provide a useful marker for functional studies of LHb neurons that preferentially innervate systems that do not have dopaminergic modulation as their primary effect.

## Materials and Methods

### Animals

All mouse strains were maintained on a C57BL/6 genetic background. The Cre and Flp driver lines for the identification of the neurotransmitter phenotypes of GABAergic and glutamatergic neurons were: *Gad2^IRES-Cre^* (*Gad2^tm2(cre)Zjh/J^*, Jax #010802; Taniguchi et al., 2011), *Slc17a6^IRES-Cre^* (*Vglut2^IRES-Cre^*, *Slc17a6^tm2(cre)Lowl/J^*, Jax 016963; Vong et al., 2011), and *Vglut2^IRES-FlpO^* (*Slc17a6^tm1.1(flpo)Hze/J^*; Daigle et al., 2018). These are available from The Jackson Laboratory and are referred to here as *Gad2^Cre^*, *Vglut2^Cre^* and *Vglut2^FlpO^*. Two Cre-inducible transgenic reporter lines were used for the genetic identification of neurotransmitter phenotypes: *Ai6*, expressing a ZsGreen reporter [*Gt(ROSA)26Sor*^*tm6(CAG-ZsGreen1)Hze/J*^, Jax #007906; Madisen et al., 2010], and *Ai75*, expressing a nuclear tdTomato reporter [*Gt(ROSA)26Sor*^tm75.1(CAG-tdTomato^*^)*Hze/J*^, Jax #025106; Quina et al., 2017]. Intersectional transgenic identification of neurons which express, or have a developmental history of expressing, both GAD2 and VGluT2 was performed with the Cre/Flp-dependent tdTomato reporter strain *Ai65* [*Gt(ROSA)26Sor*^*tm65.1(CAG-tdTomato)Hze/J*^, Jax #021875; Madisen et al., 2015]. To generate triple-transgenic mice, the *Gad2^Cre^* and *Vglut2^FlpO^* strains were interbred to generate compound heterozygotes; these in turn were bred to *Ai65* heterozygous or homozygous mice to produce *Gad2^Cre^*/*Vglut2^FlpO^*/*Ai65* (*Gad2-Vglut2^tdT^*) mice. *Vglut2^FlpO^* and *Ai75* mice were obtained as gifts from Hongkui Zeng, Allen Institute for Brain Science. Male Sprague Dawley Norway rats, weight 250–274 g, were obtained from a commercial vendor (Envigo).

### Anterograde tract tracing: viruses

Anterograde tracing was performed using adeno-associated virus (AAV), including Cre-activated (Flex) and Cre-silenced (Fas) vectors (“Cre-on/Cre-off” system). Viral stocks were prepared at the University of Pennsylvania Gene Therapy Program Vector Core (http://www.med.upenn.edu/gtp/vectorcore/). For Cre-dependent labeling of cell bodies and axons we used AAV pCAG.FLEX.tdTomato.WPRE (“FLEX-tdT,” Addgene plasmid #51503) or AAV pCAG.FLEX.EGFP.WPRE (Addgene plasmid #51502; Harris et al., 2012). Enhanced labeling of axon terminals was performed by Cre-dependent viral expression of a synaptophysin-EGFP fusion protein (sypGFP). The plasmid pCAG.Flex.sypEGFP.WPRE (“FLEX-sypGFP”) was constructed by replacing the EGFP moiety of pCAG-FLEX-EGFP-WPRE with the sypEGFP construct from phSyn1(S)-FLEX-tdTomato-T2A-SypEGFP-WPRE (Addgene #51509) by Julie Harris, Karla Hirokawa, and Hong Gu of the Allen Institute for Brain Science (gift of Julie Harris). In most experiments the tdTomato axonal tracer and the sypGFP synaptic tracer viruses were co-injected. Cre-inactivated expression was performed with pAAV-Ef1a-FAS-tdTomato-WPRE (“FAS-tdT,” Addgene #37092; Saunders et al., 2012). In most cases, FAS-tdT was co-injected with the FLEX-GFP or FLEX-sypGFP virus. Viral methods for optogenetic electrophysiological experiments are described below. All viruses used were AAV capsid strain 1.

### Anterograde and retrograde tracing: specific methods

The targeted coordinates for each anterograde and retrograde tracing injections, based on a standard atlas (Paxinos and Franklin, 2001), appear in the figure legends. The detailed methods used here for anterograde tract tracing with iontophoretic injection of AAV have been published in conjunction with the Allen Mouse Brain Connectivity Atlas (Harris et al., 2012; Oh et al., 2014). Animals were fixed by transcardial perfusion with 4% paraformaldehyde at 14–21 d after injection, and processed as described below.

Retrograde tracing was performed using a 1% solution of cholera toxin B subunit (CTB; List Biological Labs), injected using standard stereotaxic coordinates (Paxinos and Franklin, 2001). Small focal injections were performed by iontophoresis using a pulled glass pipette, using published methods (Harris et al., 2012). In brief, the pipette was positioned using a stereotaxic frame, and current was delivered with a Midgard Precision Current Source (Stoelting). The current was set to 3 μA, and was alternated for 7 s on and 7 s off for 10 min, followed by a 5-min rest period before the needle was retracted. Brain tissue was harvested and processed as described below 7–14 d following injection.

### Tissue preparation and immunofluorescence

Mouse brain tissue was prepared by fixation via transcardial perfusion with 4% paraformaldehyde. Brains were then removed and equilibrated in graded sucrose solutions, frozen at −80°C in OCT solution, and cryosectioned at 25 μm for fluorescence/immunofluorescence imaging. Tissue processed in this way was suitable for imaging of endogenous protein fluorescence, immunofluorescence, and fluorescence *in situ* hybridization (FISH). Rat brains used in FISH experiments were harvested from unperfused animals, frozen at −80°C in OCT solution, and cryosectioned at 25 μm. FISH protocols for perfused mouse brains and unperfused rat brains both included paraformaldehyde fixation of sectioned tissue on slides. Primary antisera used included: rabbit anti-parvalbumin (PV-28, Swant, RRID:AB_2315235), rabbit anti-somatostatin (SST; T-4103, Peninsula, RRID:AB_518614), goat anti-choline acetyltransferase (ChAT; AB144P, EMD Millipore, RRID:AB_2079751), and rabbit anti-tryptophan hydroxylase (ABN60, EMD Millipore, RRID:AB_10806898). The endogenous fluorescence of GFP and tdTomato reporters was enhanced with goat anti-GFP (ab6673, Abcam, RRID:AB_305643); rabbit anti-GFP (A11122, Thermo Fisher, RRID:AB_221569); rabbit anti-red fluorescent protein (600-401-379, Rockland Immunochemicals, RRID:AB_2209751).

### FISH

FISH was performed with the RNAscope Multiplex Fluorescent V2 kit, according to the manufacturer’s instructions (Advanced Cell Diagnostics). The mouse probes used included: GAD2 (catalog #415071), and Slc17a6 (VGluT2, #319171). The rat probes used included GAD1 (#316401), GAD2 (#435801), Slc32a1 (VGAT, #424541), and Slc17a6 (#317011). As described in Results, we noted surprising interspecies differences in the expression of GAD1 and GAD2 in the LHb, the former being expressed only in the rat, and the latter only in the mouse. For this reason, we considered that the probes in one species might be incorrectly assigned to the genome. The mouse GAD1 probe set used in the Allen Atlas is designed to the mRNA sequence NM_008077, located on mouse Chr2, and the rat GAD1 RNAscope probe set is designed to NM_017007, on rat Chr3; the flanking gene structure shows that these transcripts clearly represent orthologous genes. The mouse GAD2 probe set used in the Allen Atlas, and also the GAD2 RNAscope probe set, are designed to mRNA sequence NM_008078, on mouse Chr2. The rat GAD2 RNAscope probe set is designed to NM_012563, on rat Chr17, which clearly represents the orthologous gene. Thus, the correspondence of the GAD1 and GAD2 probes to the mouse and rat genome is correct.

### Electrophysiology

For optogenetic electrophysiological experiments in which post-synaptic activity was recorded in areas receiving LHb afferents, injections were performed with the conditional Channelrhodopsin2-expressing virus: AAV1.EF1a.DIO.hChR2(H134R)-EYFP.WPRE.hGH (“DIO-ChR2-EYFP,” Addgene #20298). This vector was mixed with an equal titer of AAV FLEX-sypGFP to intensify the presynaptic fluorescent signal in tissue slices. *Gad2^Cre^* mice of both sexes were injected in the LHb bilaterally at the coordinates: AP −1.40, ML ±0.35, DV 2.65. Mice were anesthetized with isoflurane then perfused transcardially with ice-cold low Na^+^ saline (“slicing solution,” which included the following: 252 mm sucrose, 2 mm KCl, 2 mm MgCl_2_, 2.6 mm CaCl_2_, 1.2 mm NaH_2_PO_4_, 26 mm NaHCO_3_, and 15 mm glucose, with the pH adjusted to 7.4 ± 0.5 and the osmolarity to 310 ± 5 mOsm). The brain was dissected, and after trimming the brainstem and midbrain were embedded in low-gelling agarose. Coronal slices were then cut at 200 or 250 μm in ice-cold slicing solution continuously oxygenated with 95% O_2_/5% CO_2_. After cutting, slices were immediately returned to the same solution and maintained in a warm bath (28 ± 0.5°C) for recovery. After 30 min, they were transferred into normal ACSF, with the same components as slicing solution except for the replacement of sucrose by 126 mm NaCl. Slices were then kept at room temperature until recording.

For whole-cell patch recording, slices were placed in a submerged recording chamber and continuously perfused with oxygenated ACSF at a rate of 1–2 ml/min. Recording was done at 31 ± 1°C. The glass pipettes for patch recording had resistances of 5–8 MΩ after being filled with an internal solution containing the following: 132 mm potassium gluconate, 10 mm HEPES, 2 mm MgCl_2_, 5 mm EGTA, 0.5 mm CaCl_2_, 4 mm ATP, 0.5 mm GTP, and 5 mm phosphocreatine, with the pH adjusted to 7.4 ± 0.5 and the osmolarity to 285 ± 5 mOsm. Slices were visualized using fluorescence optics to identify areas with dense input of LHb fibers, then recordable cells in these areas were visualized under infrared Nomarski optics using the 40× water-immersion objective of an upright microscope (Olympus, BX51WI). The patch electrode was advanced toward the target cells by a micromanipulator (Sutter) and a gigaohm seal was established, typically by a small negative pressure, with the membrane ruptured by gentle suction and/or zap pulses. Signals were amplified (MultiClamp 700A, Molecular Devices), digitized (D1322, Axon), and stored for offline analysis. EPSCs were recorded under voltage-clamp while cells were held at −70 mV. pClamp 10 software (Molecular Devices) was used for data acquisition and analysis. Optogenetic stimulation of slice preparations was delivered from a 473-nm blue laser (Cobolt) to the tissue via a 200-μm optical fiber. Light pulses of 10 or 15 ms were triggered via TTL signals from the pClamp software interface. The tip of the optical fiber was submerged in the ACSF in the recording chamber at a tip distance of ∼1.5 mm from the recording site. The light intensity was 1–3 mW/mm^2^ at the recording surface. Cells that exhibited EPSCs in response to the light stimulus were stimulated at intervals of 2 s for 20–30 cycles, and consecutive traces were collected for analysis. The stimulation protocol was then repeated following addition of the AMPA glutamate receptor blocker CNQX (20 μm) to the recording bath. Most recorded cells that exhibited EPSCs maintained an adequate seal long enough for the stimulation protocol to be repeated in the presence of CNQX at a holding potential of –70 mV. The EPSC frequency was analyzed for 20 consecutive traces, and EPSC events were counted using an amplitude threshold of 10 pA. The EPSC frequency during the 15-ms photostimulation interval was compared with the spontaneous EPSC frequency averaged over 45 ms of the interstimulus interval immediately preceding light onset, normalized for the longer sampling period. A subset of same neurons was also analyzed for light-evoked currents at holding potentials of –40 and +40 mV in the presence of CNQX to detect GABA-mediated currents that might only be identified at holding potentials far from the reversal potential for chloride ions. Results were analyzed by two-way repeated measures ANOVA for the effect of light stimulus and the effect of CNQX.

## Results

The LHb receives diverse inputs from the ventral forebrain and diencephalon, and in turn produces a complex network of output pathways. Partially distinct outputs have been described for the principal subnuclei of the LHb, with the medial subnucleus projecting predominantly to the raphe, and the lateral subnucleus accounting for most projections to the RMTg. However, there are no known molecular markers that distinguish these areas. In general, little is known about neuronal diversity in the LHb, or the functional significance of such diversity.

### Expression of GABAergic and Glutamatergic markers in the mouse LHb

**Figure 1.**
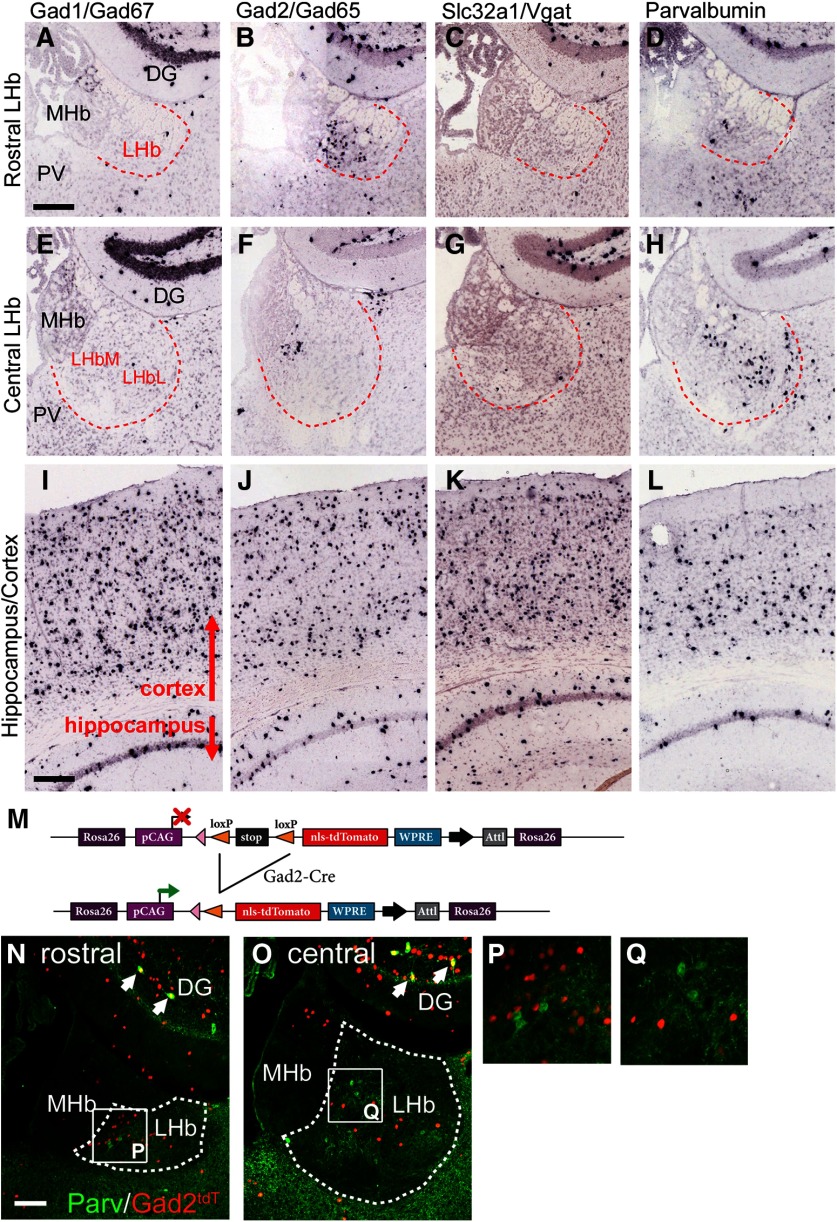
Markers of GABAergic phenotypes in the LHb. ***A–H***, Expression of mRNA for the GABAergic markers GAD1, GAD2, Slc32a1/VGAT, and parvalbumin in the rostral (bregma -1.1 to -1.4) and central (bregma -1.5 to -1.8) habenula were examined in the Allen Brain Atlas (Ng et al., 2009). GAD2-expressing neurons are most dense in the rostral LHb (***B***). In the central LHb (***F***), they are often clustered in the ventromedial part of the nucleus but may be found throughout. GAD1-expressing and VGAT-expressing neurons are rarely seen. Parvalbumin-expressing neurons in the LHb are distributed differently from the GAD2 cells, predominantly in the central LHb in LHbL (***H***). GABAergic neurons in the adjacent dentate gyrus express each of these markers. ***I–L***, The four markers show the expected distribution in the cortex and hippocampus, where they all mark inhibitory interneurons. ***M***, Transgenic strategy for genetic marking of GAD2-expressing neurons. Reporter mice with a Cre-dependent, nuclear-localized tdTomato expression cassette were interbred with *Gad2^Cre^* mice, yielding *Gad2^tdT^* mice. ***N–Q***, Gad2^tdT^ expression combined with parvalbumin immunofluorescence in the LHb. Arrows in ***N***, ***O*** indicate co-localization of Gad2^tdT^ and PV in GABAergic neurons in the DG (yellow). These markers do not colocalize at the cellular level in the LHb. ***P***, ***Q***, *In situ* hybridization data are derived from Allen Brain Atlas case numbers: GAD1, 79556706; GAD2, 79591669; VGAT, 72081554; and parvalbumin, 79556738. DG, dentate gyrus; LHb, lateral habenula; MHb, medial habenula; PV, paraventricular nucleus (of thalamus). The dashed red line in ***A–H*** and the dashed white line in ***N***, ***O*** indicates the border of the LHb. Scale bars: 200 μm (***A***, ***I***) and 100 μm (***N***).

One potential functional subtype of LHb neurons are the “GABAergic” neurons that have been reported in the rat LHb (Brinschwitz et al., 2010; Zhang et al., 2016; Zahm and Root, 2017). GABAergic neurons throughout the brain are generally characterized by the co-expression of two biosynthetic enzymes, GAD1 and GAD2, and the VGAT. In order to determine whether a population of GABAergic neurons exists in the mouse LHb, we examined expression of these GABAergic markers in a gene expression database that has previously been used to assess habenula gene expression (Quina et al., 2009). Database *in situ* hybridization data revealed a population of LHb neurons that express GAD2, but surprisingly expressed neither GAD1 nor VGAT at detectable levels (Fig. 1). These LHb GAD2 neurons are concentrated in the rostral (Fig. 1*B*), and the ventromedial corner (Fig. 1*F*) of the LHb. As expected, in the database cases examined, GABAergic neurons in the cortex and hippocampus expressed all three markers at robust levels (Fig. 1*I–K*). Another identifiable subpopulation of LHb neurons express the neuropeptide parvalbumin (Pozzi et al., 2014), a peptide which, in the neocortex, is associated with GABAergic neurons. However, neurons expressing PV mRNA were sparse in the rostral and ventromedial LHb, where most of the GAD2 neurons appear (Fig. 1*D*,*H*), suggesting that these are different cell types. In the cortex and hippocampus, the distribution of the PV mRNA signal was similar to that of the other GABAergic markers (Fig. 1*L*).

The wide expression of GAD2 protein in the neuropil of most brain regions makes clear immunofluorescence labeling of GABAergic cell bodies difficult. For this reason, to localize the LHb GAD2 neurons for further studies, we took a transgenic approach. Mice bearing a *Gad2^Cre^* allele were interbred with the transgenic line *Ai75*, which supports Cre-dependent expression of a nuclear-localized tdTomato reporter (tdT^nuc^; Fig. 1*M*). This resulted in robust tdT^nuc^ expression in a pattern similar to GAD2 mRNA in the LHb, as well as in GABAergic neurons throughout the neural axis. LHb GAD2 neurons expressing tdT^nuc^ showed no overlap with cells expressing PV immunoreactivity (Fig. 1*N–Q*).

**Figure 2.**
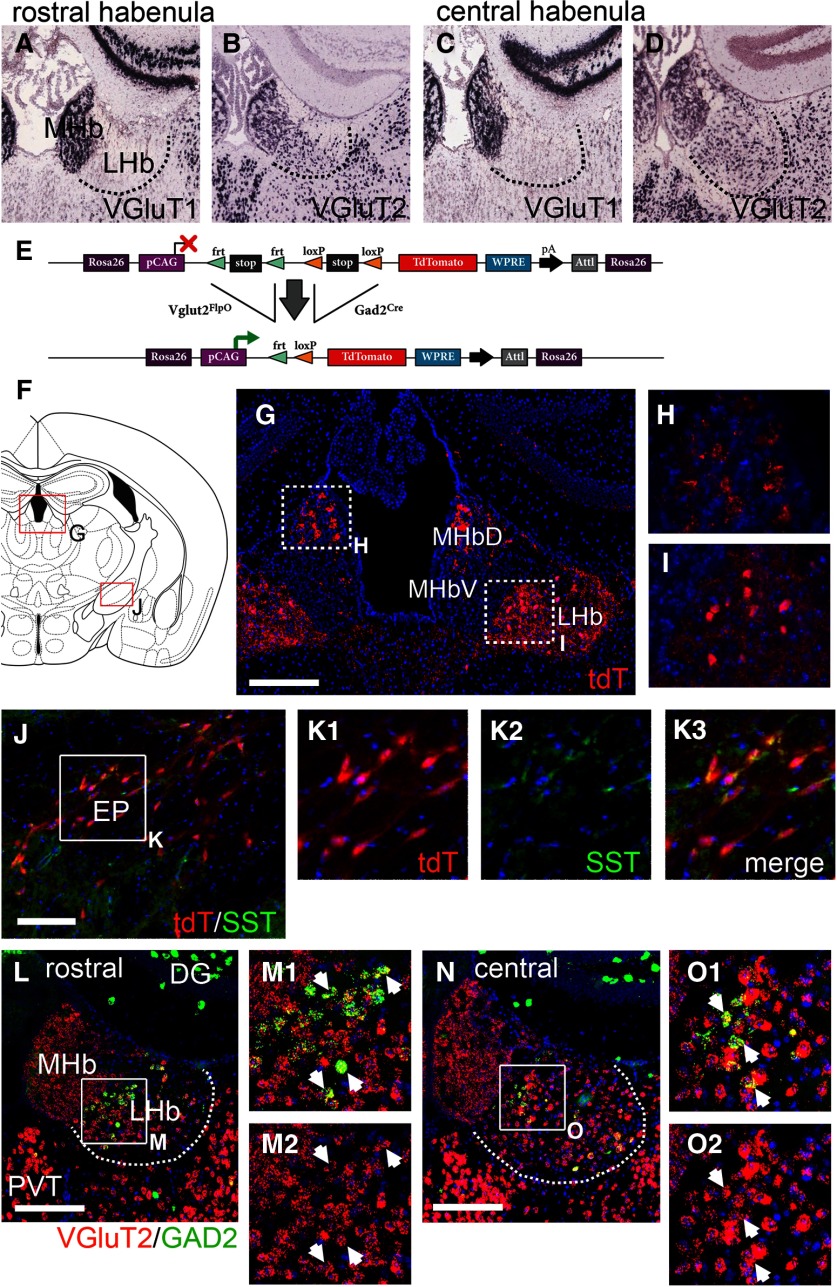
LHb GAD2-expressing neurons express VGluT2. ***A–D***, Expression of the glutamatergic markers Slc17a7 (VGluT1) and Slc17a6 (VGluT2) in the habenula. The LHb expresses predominantly the subcortical transporter VGluT2, while the MHb expresses both transporters. ***E***, Transgenic strategy for intersectional marking of Gad2^Cre^ and Vglut2^Flp^ co-expressing neurons. The tdTomato marker labels both the cell bodies and axons of *Gad2/Vglut2^tdT^* mice. ***F***, Standard atlas view at bregma -1.22 showing the location of detailed images for the rostral LHb and EP (also called MGP and GPi; Paxinos and Watson, 1998). ***G–I***, Expression of tdTomato in the rostral habenula of *Gad2/Vglut2^tdT^* mice. Confocal imaging in ***H*** shows that tdT expression is limited to rosettes of afferent fibers in the MHbD, whereas ***I*** shows both labeled cell bodies and afferent fibers in the LHb. ***J–K***, Expression of tdT and SST in the EP of *Gad2/Vglut2^tdT^* mice. The EP is a likely source of some of the LHb afferent fibers seen in ***G***. ***L–O***, FISH for VGluT2 and GAD2 in the rostral (***L***) and central (***N***) habenula. Confocal imaging in ***M***, ***O*** shows co-localization of the VGluT2 and GAD2 signals. Arrows indicate examples of discrete co-expressing neurons. *In situ* hybridization data in ***A–D*** are derived from Allen Brain Atlas case numbers: VGluT1, 70436317 and VGluT2, 73818754. DG, dentate gyrus; EP, entopeduncular nucleus; LHb, lateral habenula; MHb, medial habenula (*D*, dorsal, *V*, ventral); PV, paraventricular nucleus (of thalamus). The dashed white line in ***L***, ***N*** indicates the border of the LHb. Scale bars: 200 μm (***G***), 100 μm (***J***), and 200 μm (***L***, ***N***).

Since VGAT is necessary for the use of GABA as a synaptic neurotransmitter, these results led us to question whether the LHb GAD2 neurons actually use GABA as their principal fast neurotransmitter. The majority of LHb neurons are glutamatergic, and predominantly express the subcortical vesicular glutamate transporter gene Slc17a6/Vglut2, not the cortical transporter Slc17a7/Vglut1 (Fig. 2*A–D*). In order to determine whether LHb GAD2 neurons could be a subset of excitatory neurons, we first took an intersectional transgenic strategy to identify neurons that co-express GAD2 and VGluT2, using triple-transgenic mice with *Gad2^Cre^* and *Vglut2^FlpO^* transgenes, and a Cre/Flp-dependent reporter allele targeted to the *Gt-Rosa(26)* locus which allows conditional expression of tdT in any cell type (Fig. 2*E*; Materials and Methods). Reporter expression in the habenula was more extensive than expected (Fig. 2*F*,*G*). However, much of the LHb signal was accounted for by afferent fibers that labeled the neuropil rather than being associated with cellular profiles. Afferent tdT-expressing fibers were observed in the dorsal medial habenula (MHbD) and extensive tdT labeling was also observed in the LHb (Fig. 2*G*). Confocal imaging showed that in the MHbD tdT signal appeared to be associated with neuropil (Fig. 2*H*), consistent with the absence of GAD2 mRNA in the MHbD. In contrast, in addition to diffuse fiber labeling, tdT-labeled cell bodies were evident in the ventromedial LHb (Fig. 2*I*), consistent with the location of the LHb GAD2 neurons identified by *in situ* hybridization. One likely source for tdT-labeled LHb afferents are glutamate/GABA co-releasing neurons recently identified in the entopeduncular nucleus (EP; also known as the globus pallidus internus and the medial globus pallidus), which also express SST (Shabel et al., 2014; Meye et al., 2016; Wallace et al., 2017). Indeed, a population of tdT-expressing cells was observed in the EP of *Gad2^Cre^*/*Vglut2^FlpO^* tdT mice (Fig. 2*J*), and many of these neurons expressed SST (Fig. 2*K*). LHb-projecting VTA neurons that co-release glutamate and GABA are also likely to contribute to these tdT-labeled afferents (Root et al., 2014a,b). EP and VTA dual-expressing neurons projecting to the LHb appear to sequester GABAergic and glutamatergic markers in discrete synaptic vesicles (Root et al., 2018). However, these studies of LHb afferents do not address the neurotransmitter identity of the LHb neurons themselves.

Intersectional reporter activation by *Gad2^Cre^*/*Vglut2^FlpO^* is a “fate mapping” experiment in which reporter expression could in principle be activated by transient expression of *Gad2^Cre^* or *Vglut2^FlpO^* during development, with persistent activation of the reporter allele. Thus, expression of the tdT reporter does not prove cellular co-expression of GAD2 and VGluT2 mRNA in the adult brain. In order to determine whether GAD2 and VGluT2 co-expression persist in the LHb of adult mice, we used double-label FISH for these markers (Fig. 2*L–O*). VGluT2 was consistently expressed in the LHb GAD2-expressing neurons, no cells were observed expressing GAD2 alone.

### Expression of GABAergic markers in the rat LHb

**Figure 3.**
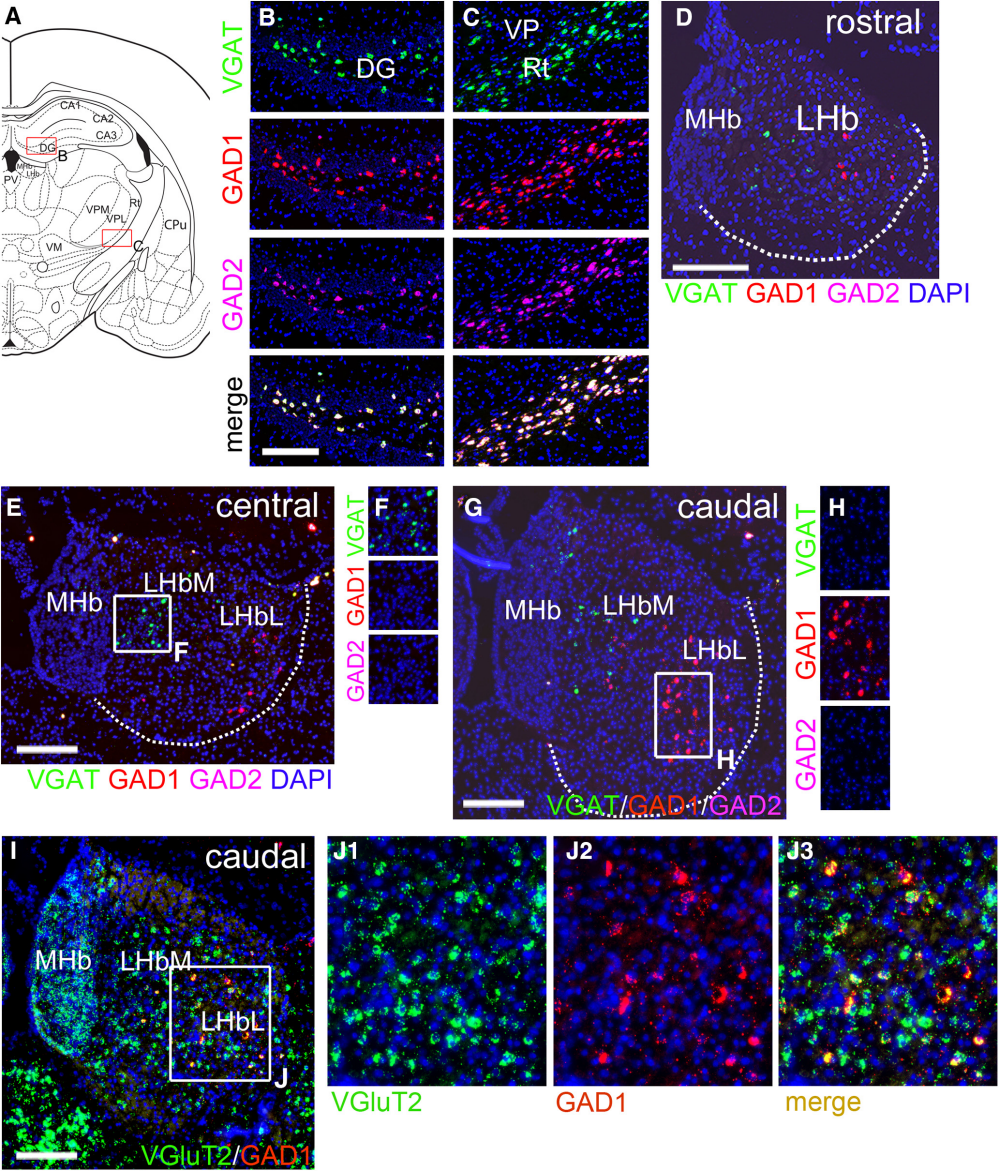
Expression of GABAergic markers in the rat LHb. FISH was performed for a combination of the GABAergic markers VGAT, GAD1 and GAD2, and for GAD1 plus VGluT2. Sections were selected at rostrocaudal levels that had the largest population of cells expressing the markers shown. ***A–C***, Co-expression of GABAergic markers in “typical” GABAergic neurons. ***A***, Standard atlas view at bregma -3.14 showing location of detailed views (Paxinos and Watson, 1998). Expression of GABAergic markers in the dentate gyrus (***B***) and in the reticular thalamic nucleus (***C***). ***D***, Expression of GABAergic markers in the rostral habenula, at bregma -2.6. The rostral population of GAD2-expressing neurons present in the mouse are not detected in the rat. ***E***, ***F***, Expression of GABAergic markers in the central habenula, at bregma -3.2. ***G***, ***H***, Expression of GABAergic markers in the caudal habenula, at bregma -3.6. ***I***, ***J***, Expression of VGluT2 and GAD1 in the caudal habenula. GAD1-expressing neurons at all levels co-express VGluT2. DG, dentate gyrus; LHb, lateral habenula, LHbL, lateral subnucleus; LHbM, medial subnucleus; MHb, medial habenula; Rt, reticular nucleus of thalamus; VP, ventral posterior thalamus. The dashed white line in ***D***, ***E***, ***G*** indicates the border of the LHb. Scale bars: 200 μm (***B***, ***D***, ***E***, ***G***, ***I***).

Reports of the expression of GABAergic markers in the Norway rat LHb has been inconsistent, and subject to different interpretations (Zahm and Root, 2017). However, prior rat studies have not identified a large population of GAD2-expressing neurons in the rostral or ventromedial part of the LHb, as shown here in mice. For this reason, we used FISH to systematically examine the expression of VGAT, GAD1, and GAD2 in the Norway rat habenula. Triple-label FISH readily identified co-expression of these three markers in GABAergic neurons of the dentate gyrus and reticular thalamic nucleus in the same sections used to visualize these markers in the LHb (Fig. 3*A–C*). A few weakly VGAT-expressing neurons were detected in LHbM (Fig. 3*D–F*); these were most abundant in the central part of the LHb with respect to the rostrocaudal axis. GAD1 and GAD2 expression could not be detected in the VGAT-expressing cells (Fig. 3*F*). A scattered population of GAD1-expressing neurons were detected in LHbL (Fig. 3*D–H*); these were most abundant in the central to caudal part of the LHb (Fig. 3*G*). There was no evidence of VGAT expression in the GAD1-expressing neurons (Fig. 3*H*). Expression of GAD2 was not detected in the rat LHb; in particular, GAD2 neurons were absent from the rostral LHb (Fig. 3*D*), where they are especially abundant in the mouse.

Because the GAD1-expressing neurons in the rat LHb do not exhibit detectable levels of VGAT, we hypothesized that they would co-express VGluT, like the GAD2-expressing neurons in the mouse LHb. Indeed, double-label FISH for GAD1 and VGluT2 in the rat LHb showed consistent co-expression in the GAD1-expressing neurons (Fig. 3*I*,*J*). To more accurately estimate the fraction of GAD1-expressing cells that also expressed VGluT2, we counted all GAD1-expressing cells in both hemispheres of eight sections taken at 100-μm intervals through the central and caudal LHb. Only neurons that contained a DAPI-stained nucleus within the plane of section were counted. Of 231 GAD1^+^ cells counted, 226 (98%) had detectable VGluT2 mRNA expression.

### Anterograde tracing of LHb GAD2 neuron projections to the mesopontine raphe and tegmentum

**Figure 4.**
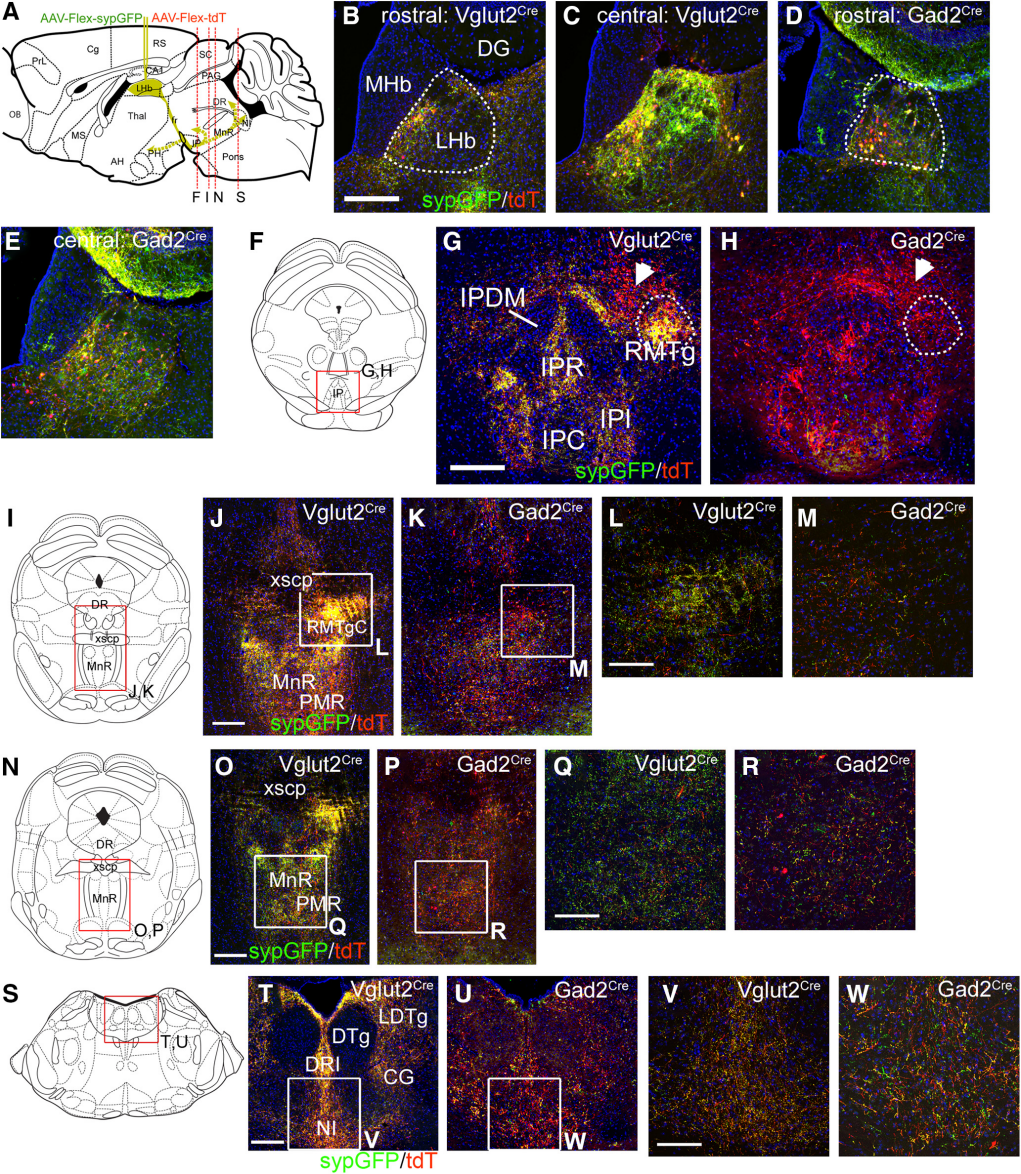
Specific projections of LHb GAD2-expressing and VGluT2-expressing neurons. ***A***, Strategy for Cre-dependent labeling of LHb efferents using a mixture of a synapse-targeted GFP reporter virus, AAV-Flex-sypGFP, and a cytoplasm/axon-targeted tdTomato reporter virus, AAV-Flex-tdT. ***B–E***, Reporter expression in the injected area of the LHb of *Vglut2*^*Cre*^ (***B***, ***C***) and *Gad2*^*Cre*^ (***D***, ***E***) mice. Injection coordinates were: AP -1.64, ML 0.25, DV 2.56, and AP -1.3, ML 0.3, DV 2.6, respectively. The *Gad2^Cre^* injection was positioned to maximize labeling of Gad2-expressing neurons. Overall, because of the wider expression of VGlutT2, more cells, and consequently more efferent fibers, are labeled in *Vglut2^Cre^* than in *Gad2^Cre^* mice. ***F–H***, LHb fibers at the level of the IP and RMTg, corresponding to bregma -1.88 in a standard atlas (Paxinos and Franklin, 2001). Vglut2^Cre^-driven expression heavily labels synapses in the RMTg (***G***, circle), but synapses from GAD2-expressing LHb neurons are sparse at this level (***H***, circle). Arrows indicate fibers of passage (red) to more caudal areas, which have similar levels of labeling. ***I–M***, LHb fibers and synapses at the level of the DR and MnR (bregma -4.36). The area designated RMTgC is identified as part of the anterior tegmental nucleus in standard atlases, but has been shown to be part of the RMTg complex (Quina et al., 2015). ***N–R***, LHb fibers and synapses in the caudal MnR (bregma -4.60). ***S–W***, LHb fibers and synapses in the dorsal tegmentum, including the LDTg and NI (bregma -5.34). Synapses from GAD2-expressing neurons are abundant at this level. CGPn, pontine CG; DG, dentate gyrus; DRI, dorsal raphe, inferior part; DTg, dorsal tegmentum; IPC, interpeduncular nucleus, caudal; IPDM, interpeduncular nucleus, dorsomedial; IPI, interpeduncular nucleus, intermediate; IPR, interpeduncular nucleus, rostral; LDTg, laterodorsal tegmental nucleus; LHb, lateral habenula; MHb, medial habenula; MnR, median raphe; NI, nucleus incertus; PMR, paramedian raphe; RMTg, rostromedial tegmental nucleus (*C*, caudal part); xscp, decussation of the superior cerebellar peduncle. The dashed white line in ***B***, ***D*** indicates the border of the LHb. Scale bars: 200 μm (***B***, ***G***, ***J***, **O**, ***T***) and 100 μm (***L***, ***Q***, ***V***).

LHb neurons are known to project caudally to the VTA/RMTg, the median and DR, and the pontine tegmentum, and also rostrally to the hypothalamus (Herkenham and Nauta, 1979; Bernard and Veh, 2012; Gonçalves et al., 2012; Quina et al., 2015). However, no markers have been described for subsets of LHb neurons that discriminate between these pathways. Having established that the mouse LHb GAD2 neurons are a subset of glutamatergic neurons, rather than GABAergic in the usual sense, we next asked whether these neurons were specialized in terms of their connectivity. To address this question using anterograde tracing, we used AAV-based strategies. In one such strategy, we injected a mixture of two Cre-activated AAVs, one expressing tdTomato that predominantly labeled cell bodies, axons, and fibers of passage, and the other expressing a sypGFP fusion protein that predominantly labeled presynaptic terminals (Materials and Methods). Thus, where fibers labeled with both markers run in the plane of section through an area of synaptic contact, they resemble “green beads on a red string.” This viral mixture was injected into *Vglut2^Cre^* mice, potentially labeling any LHb neuron, and *Gad2^Cre^* mice, labeling only the GAD2 subset (Fig. 4*A*). To the extent possible, cases with similar extents of LHb labeling were chosen for comparison, but injections into *Vglut2^Cre^* mice inevitably labeled more LHb neurons than injections into *Gad2^Cre^* mice, as VGlutT2 is expressed in a much larger number of cells, including the GAD2 neurons (Fig. 4*B–E*). As expected, Vglut2^Cre^-driven expression produced dense synaptic labeling in the ipsilateral RMTg, located adjacent to the interpeduncular nucleus. However, *Gad2^Cre^* mice exhibited very little synaptic labeling within the RMTg (Fig. 4*F–H*).

Prior studies have shown that neurons with RMTg-like properties (GABAergic neurons that respond to amphetamine, and project to the VTA) continue caudally in the tegmentum to the level of the decussation of the superior cerebellar peduncle (RMTgC; Quina et al., 2015). The RMTgC was also densely labeled by Vglut2^Cre^ sypGFP, but not by *Gad2^Cre^* driven expression (Fig. 4*I–M*). In the MnR/PMnR, *Gad2^Cre^*-driven expression produced prominent synaptic labeling (Fig. 4*N–R*). *Gad2^Cre^*-driven synaptic labeling was abundant in the dorsal pontine tegmentum (Fig. 4*S–W*). Particularly in the central gray (CG) and NI (Fig. 4*T–W*), synaptic labeling driven by *Gad2^Cre^* was approximately equal to that in the Vglut2^Cre^ case. Since overall labeling of the LHb was more extensive in the Vglut2^Cre^ case, this indicates that LHb GAD2 neurons have a preference for the pontine tegmentum.

**Figure 5.**
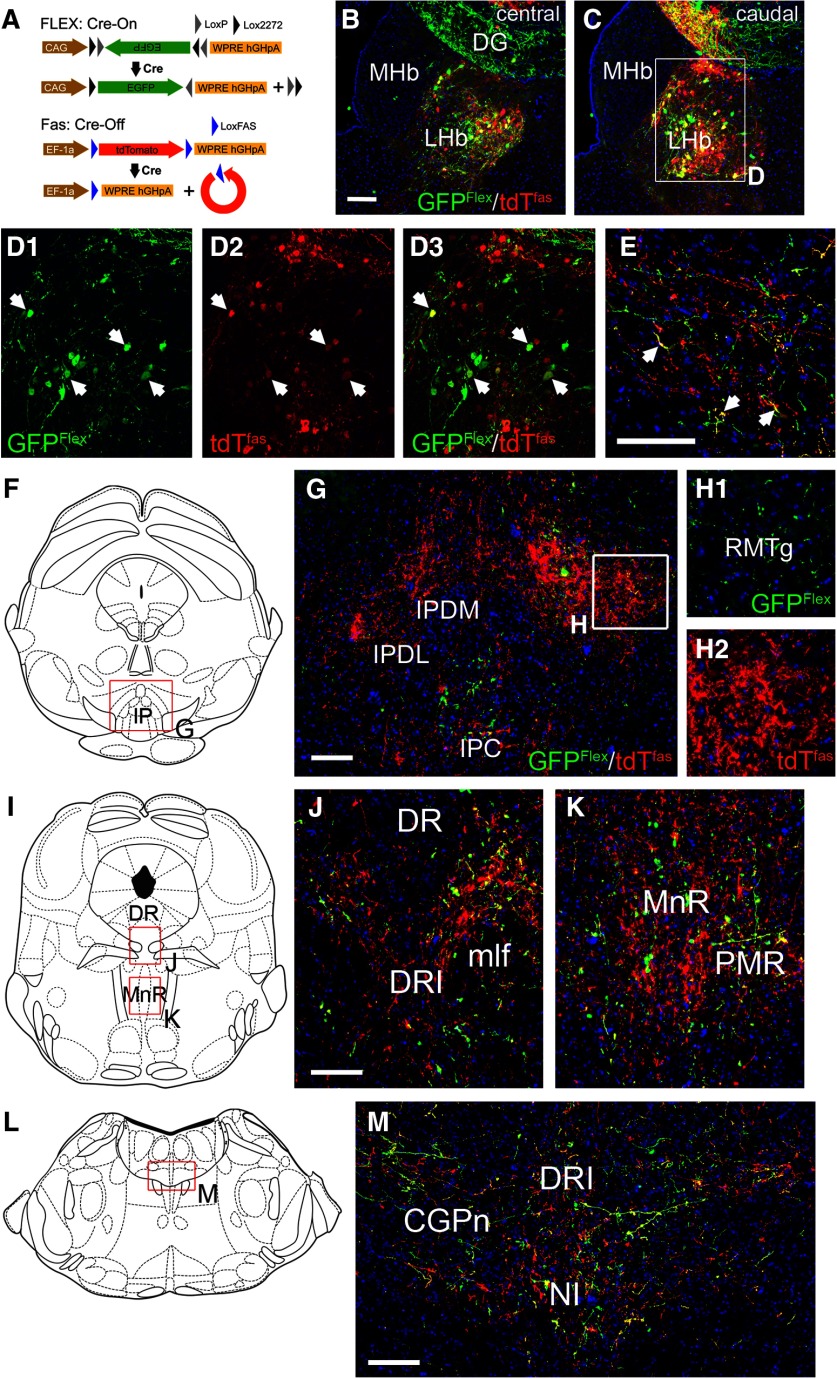
Cre-on/Cre-off mapping of LHb GAD2 neuron efferents. ***A***, Cre-on/Cre-off dual labeling strategy with a AAV-Flex-GFP virus activated by Cre recombinase and an AAV-FAS-tdTomato virus silenced by Cre. ***B***, ***C***, Image of the central (***B***) and caudal (***C***) LHb in a mouse injected with a mixture of both viruses. Some off-target labeling is observed in the dentate gyrus but these neurons do not project to any of the areas of interest. The injection coordinates were: AP -1.69, ML 0.45, DV 2.70. ***D***, Confocal images of the injected area in ***C***; arrows indicate examples of Gad2^Cre^ neurons in which the tdTomato reporter is not completely inactivated, and the markers are co-expressed, producing a yellow-green to yellow color. ***E***, Confocal image of LHb efferents in the CGPn. GAD2-negative fibers appear in red; GAD2-expressing fibers appear in green, and also in yellow due to incomplete silencing of the tdT reporter. Examples of dual-labeled (yellow) fibers are indicated by arrows. ***F–H***, LHb fibers in the IP and RMTg, corresponding to bregma -3.80 in a standard atlas. The projections of LHb GAD2-expressing neurons to the RMTg are very sparse (***H1***, green), while the projections of GAD2-negative neurons densely fill the RMTg (***H2***, red). ***I–K***, LHb efferents in the DR and MnR, and adjacent areas (bregma -4.72). ***L–M***, LHb efferents in the CGPn and NI area (bregma -5.34). CGPn, pontine central gray; DG, dentate gyrus; DR, dorsal raphe; DRI, dorsal raphe, inferior part; IPC, interpeduncular nucleus, caudal; IPDL, interpeduncular nucleus, dorsolateral; IPDM, interpeduncular nucleus, dorsomedial; LHb, lateral habenula; MHb, medial habenula; mlf, medial longitudinal fasciculus; MnR, median raphe; NI, nucleus incertus; PMR, paramedian raphe; RMTg, rostromedial tegmental nucleus. Scale bars: 100 μm (***B***, ***E***, ***G***, ***J***, ***M***).

In order to overcome the inherent variability of tract-tracing cases with separate injections into mouse strains with different Cre-drivers, we also used a Cre-on/Cre-off strategy to trace LHb efferents, combining a FLEX-GFP virus activated by Cre, and a FAS-tdTomato virus inactivated by Cre, injected into *Gad2^Cre^* mice (Fig. 5*A*; Saunders et al., 2012). In such a strategy, projections from GAD2 LHb neurons should be labeled with GFP, and those from non-GAD2 expressing LHb neurons labeled with tdT, allowing a direct comparison of these two populations of fibers in brain areas innervated by the LHb. This strategy was largely effective (Fig. 5*B*,*C*), but inactivation of FAS-tdT was not complete in all of the GFP-expressing *Gad2^Cre^* neurons, so some cell bodies (Fig. 5*D*) and terminal fibers (Fig. 5*E*) expressed both markers. This labeling strategy confirmed that the projections of the GAD2 LHb neurons to the RMTg were very sparse compared with non-GAD2 expressing cells (Fig. 5*G*,*H*). In the DR and MnR/PMnR a significant number of fibers from GAD2 LHb neurons were noted (Fig. 5*I–K*). In the pontine tegmentum, including the pontine CG (CGPn) and NI, fibers from GAD2 LHb neurons and non-GAD2 neurons were approximately equal, despite the predominance of non-GAD2 neurons in the LHb, suggesting a relative enrichment of GAD2 LHb projections to this area (Fig. 5*L*,*M*).

**Figure 6.**
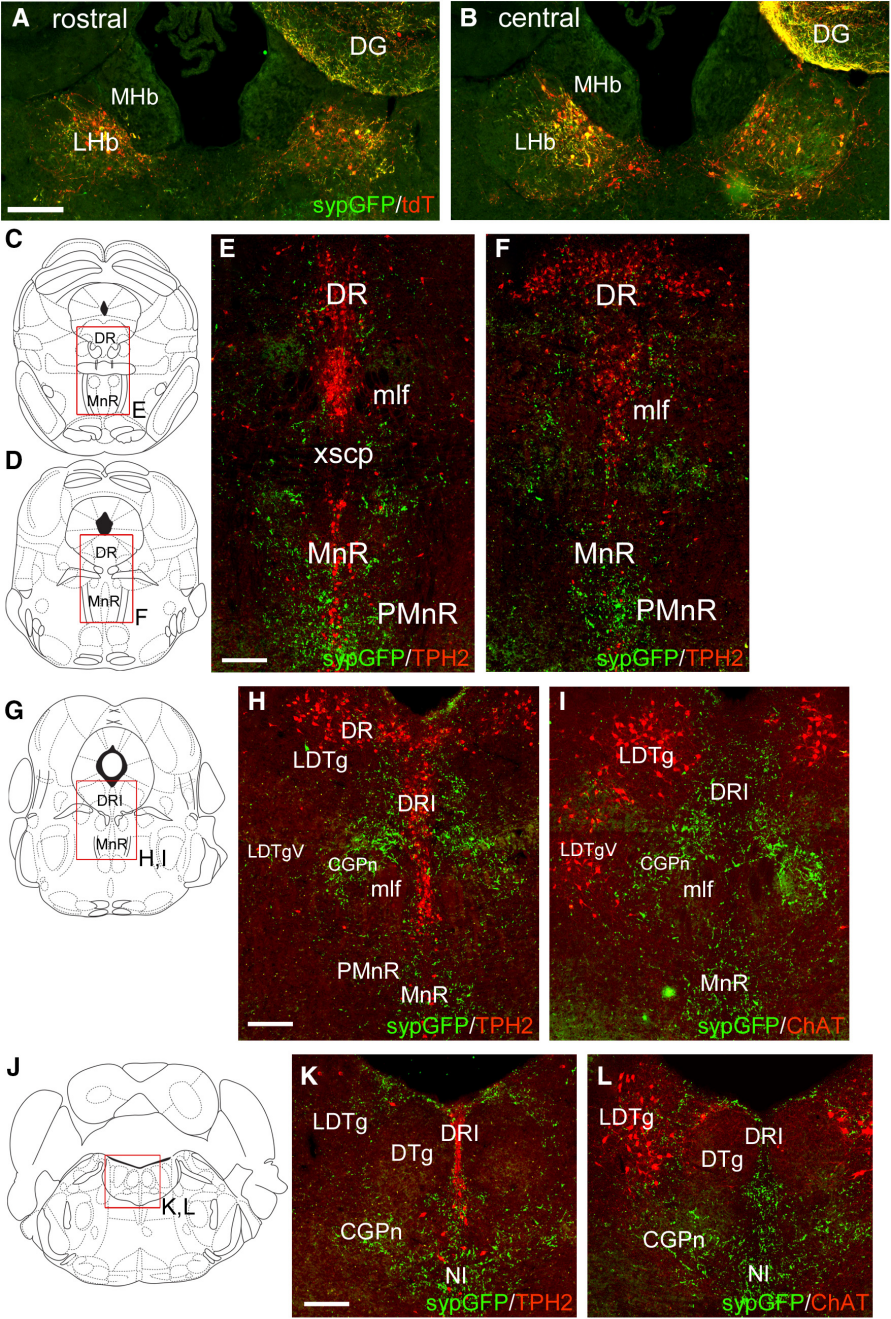
Relationship of the LHb GAD2 efferents to serotonergic neurons of the raphe and cholinergic neurons of the dorsal tegmentum. LHb efferents were traced in *Gad2^Cre^* mice, and serotonergic and cholinergic neurons were identified by immunofluorescence for Tph2 and ChAT, respectively. ***A***, ***B***, Views showing bilateral LHb injections of a *Gad2^Cre^* mouse with a mixture of AAV-Flex-sypGFP (targets synapses) and AAV-Flex-tdTomato (targets soma/axons). The injection coordinates were: AP -1.64, ML ±0.25, DV 2.60. The tdT signal is shown here to outline the extent of the injection but tdT labeling does not appear in the subsequent views. ***C–F***, Efferents of LHb GAD2 neurons in relationship to serotonergic neurons of the DR and MnR. Sections correspond to bregma -4.36 (***C***) and -4.72 (***D***) in a standard atlas. ***G–I***, Efferents of GAD2 neurons in relationship to the serotonergic (***H***) and cholinergic (***I***) neurons of the mesopontine tegmentum, at the transition from the DR to the LDTg (bregma -4.94). The area of highest synaptic density does not contain serotonergic or cholinergic cell bodies. ***J–L***, Efferents of GAD2 neurons in relationship to the serotonergic (***K***) and cholinergic (***L***) neurons of the pontine tegmentum and in the NI (bregma -5.34). CGPn, pontine central gray; DG, dentate gyrus; DR, dorsal raphe; DRI, dorsal raphe, inferior part; DTg, dorsal tegmental nucleus; LHb, lateral habenula; LDTg, laterodorsal tegmental nucleus (*V*, ventral part); MHb, medial habenula; mlf, medial longitudinal fasciculus; MnR, median raphe; NI, nucleus incertus; PMnR, paramedian raphe; xscp, decussation of the superior cerebellar peduncle. Scale bars: 200 μm (***A***, ***E***, ***H***, ***K***).

In order to better understand the relationship between the LHb GAD2 neurons and their targets in the mesopontine tegmentum, we examined the relationship between sypGFP-labeled terminals from these neurons, and two markers frequently used to identify specific populations of neurons in the tegmentum, tryptophan hydroxylase 2 (Tph2) for 5HT neurons, and ChAT for cholinergic neurons (Fig. 6). *Gad2^Cre^* mice were injected with AAV FLEX-sypGFP bilaterally (Fig. 6*A*,*B*). Labeled synapses were present but sparse in the DR, overlying 5HT neurons there (Fig. 6*C–F*). Synaptic density was higher in the MnR and PMnR than in the DR. The highest synaptic density was found in an area of the CGPn lying between the LDTg and DRI, but which contained neither cholinergic or serotonergic neurons (Fig. 6*G–I*). Strong synaptic labeling was also observed in the NI (Fig. 6*J–L*).

### LHb GAD2 neurons and the rostral projections of the LHb

**Figure 7.**
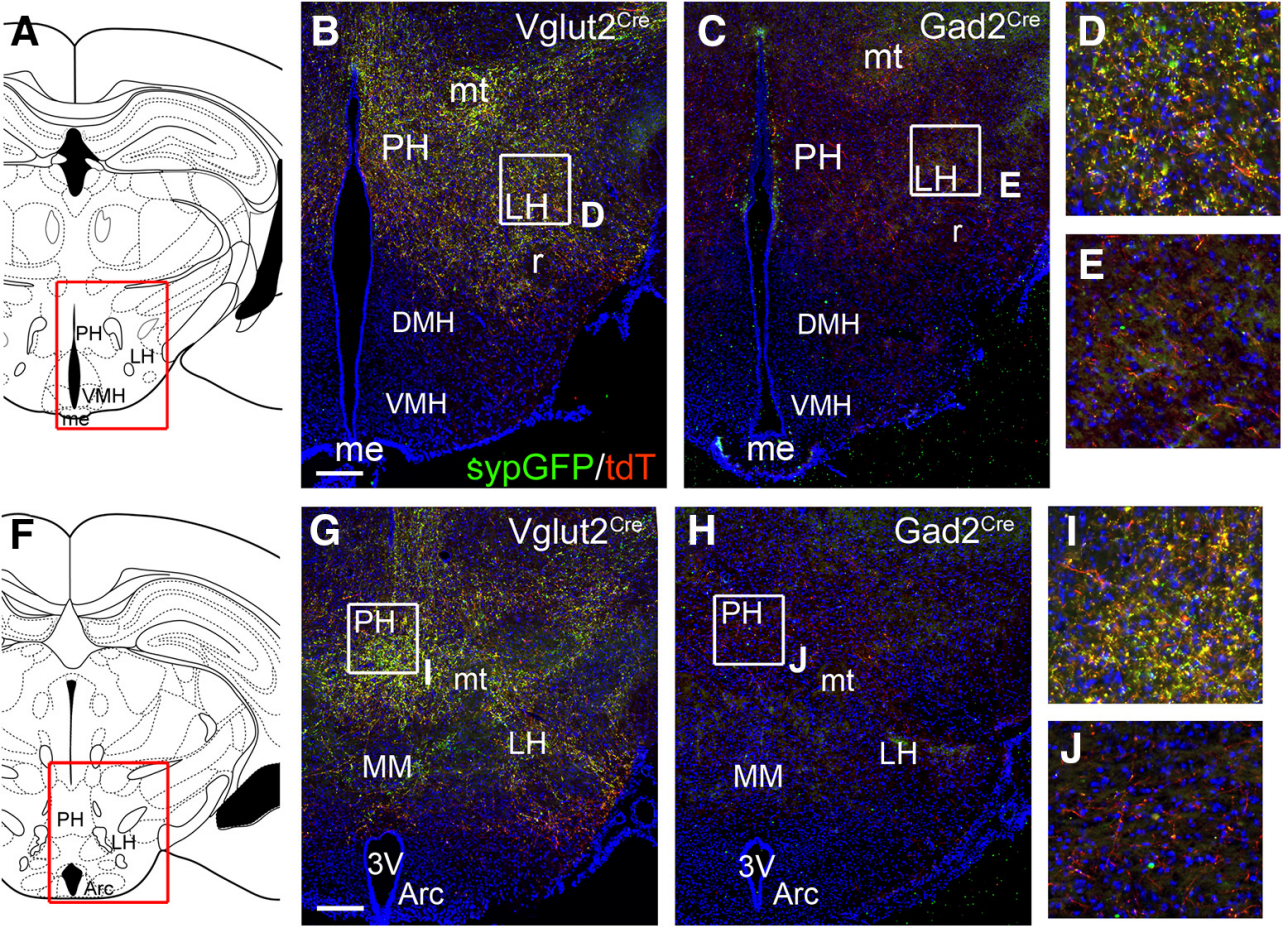
LHb projections to the hypothalamus. ***A–J***, Anterograde tracing of LHb projections to the hypothalamus using a mixture of synapse-targeted AAV-Flex-sypGFP, and cytoplasm/axon-targeted AAV-Flex-tdT. The injected area is shown in Figure 4*B–E*. ***A–E***, LHb afferents in the central hypothalamus (bregma -2.18 in a standard atlas). Dense synaptic labeling is observed in the LH of *Vglut2^Cre^* (***B***, ***D***) but not in *Gad2^Cre^* (***C***, ***E***) mice. ***F–J***, LHb afferents in the caudal hypothalamus (bregma -2.54). Dense synaptic labeling is observed in the PH of *Vglut2^Cre^* (***G***, ***I***) but not in *Gad2^Cre^* (***H***, ***J***) mice. 3V, third ventricle; Arc, arcuate hypothalamic nucleus; DMH, dorsomedial hypothalamic nucleus; f, fornix (this is labeled “r” in the figure); LH, lateral hypothalamic area; me, medial amygdaloid nucleus; MM, medial mammillary nucleus, medial part; mt, mammillothalamic tract; PH, posterior hypothalamic area; VMH, ventromedial hypothalamic nucleus. Scale bars: 200 μm (***B*** and ***G***).

In addition to its projections to the mesopontine tegmentum, the LHb sends efferent fibers to the posterior hypothalamus (PH) and lateral hypothalamus (LH). Prior retrograde tracing studies of this pathway have shown that PH-projecting LHb neurons are concentrated in the rostral 1/3 of the LHb, and at more caudal levels reside mainly in the ventromedial quadrant of the nucleus (Quina et al., 2015). Because the PH-projecting LHb neurons and the LHb GAD2 neurons are both concentrated rostrally and ventromedially, we at first considered that the GAD2-expressing neurons might account for the PH projection. To test this hypothesis, we examined *Vglut2^Cre^* and *Gad2^Cre^* mice injected in the LHb with Cre-dependent AAV expressing tract-tracing and synaptically targeted reporters (as shown in Fig. 4*B–E*) for labeling of fibers in the PH and LH (Fig. 7). Surprisingly, while labeled fibers and synaptic terminals were abundant in the PH and LH of injected *Vglut2^Cre^* mice (Fig. 7*B*,*D*,*G*,*I*), they were sparse in *GAD2^Cre^* mice (Fig. 7*C*,*E*,*H*,*J*).

### Retrograde tracing of LHb GAD2 neuron projections to the raphe and pontine tegmentum

**Figure 8.**
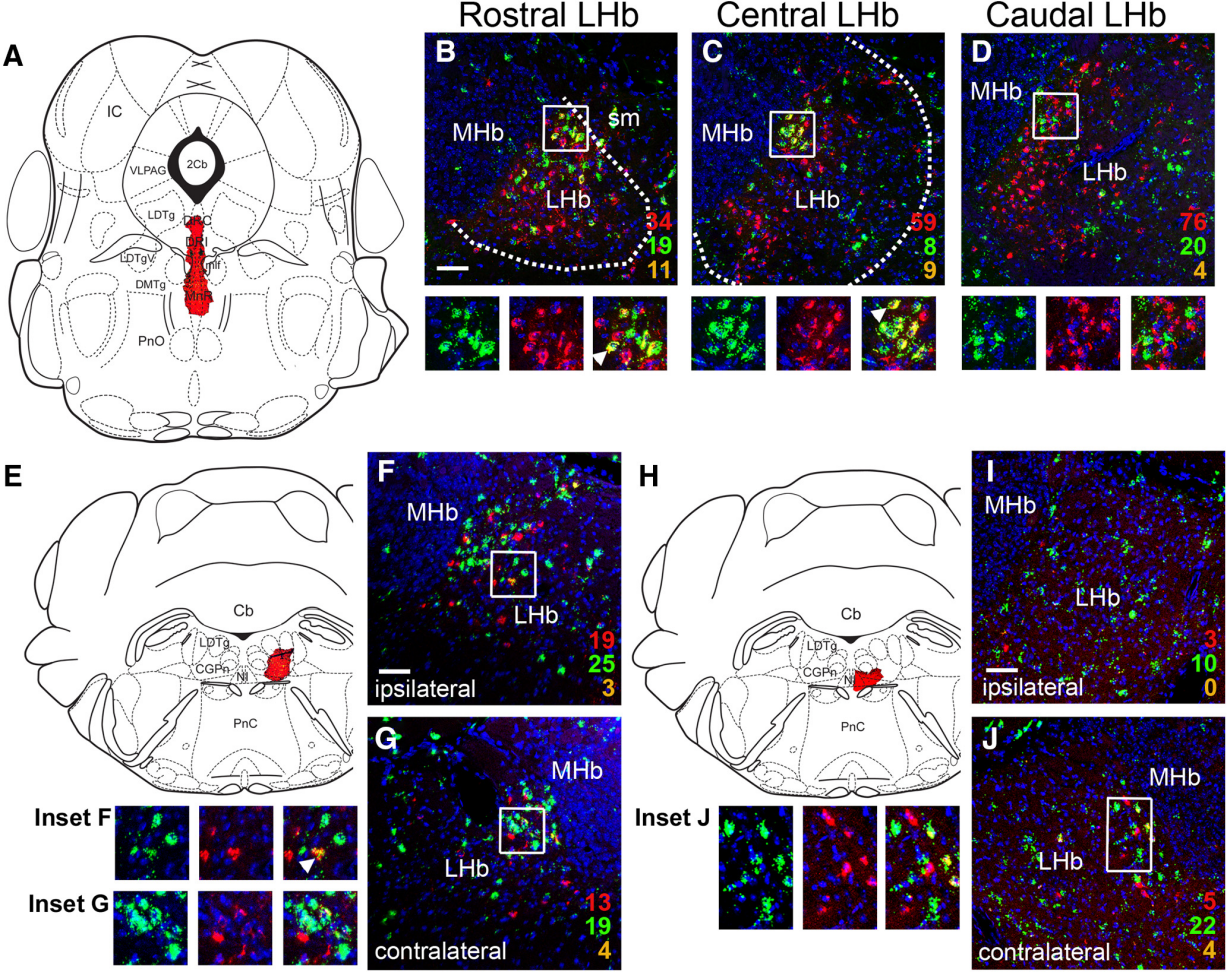
Retrograde tracing of LHb Gad2 projections to the raphe and pontine tegmentum. GABAergic neurons throughout the neural axis were genetically labeled by interbreeding *Gad2^Cre^* mice with the reporter line *Ai6*, which conditionally expresses ZsGreen (Materials and Methods). *Gad2^ZsGreen^* mice were then injected with CTB at the specified sites. Numbers indicate CTB-labeled (red), ZsGreen labeled (green), and dual labeled (yellow) neurons counted in the entire section, using confocal microscopy. Only cells with DAPI-labeled nuclei in the plane of the section were counted. Boxes indicate the area shown in the enlargements below the main figures. Arrows in the enlarged views indicate examples of dual-labeled cells. Only one example is indicated, even when multiple dual-labeled cells appear in the field of view. The number of CTB labeled neurons varied between areas approximately in proportion to the size of the injected fields. ***A***, Injection site in the caudal raphe, including DRI and caudal MnR, at bregma -4.96. Injection coordinates: AP -4.96, ML 0.0, DV 4.50. ***B–D***, ZsGreen expression and CTB retrogradely transported from the raphe in the rostral, central, and caudal LHb. ***E***, Injection site in the right lateral pontine tegmentum, including CGPn and LDTg, at bregma -5.52. Injection coordinates: AP -5.70, ML 0.40, DV 4.15. ***F***, ***G***, ZsGreen expression and CTB retrogradely transported from the pontine tegmentum in the central LHb on the ipsilateral (right, ***F***) and contralateral (left, ***G***) side. ***H***, Injection site near the midline of the pontine tegmentum, confined by fiber tracts largely to the right side, including the NI and CGPn. Injection coordinates: AP -5.52, ML 0.03, DV 4.25. ***I***, ***J***, ZsGreen expression and CTB retrogradely transported from the pontine tegmentum in the central LHb on the ipsilateral (right, ***I***) and contralateral (left, ***J***) side. LHb, lateral habenula; MHb, medial habenula; sm, stria medullaris of the thalamus. Scale bars: 50 μm (***B***, ***F***, ***I***).

Prior studies have used retrograde tracing with CTB to comprehensively map the projections of the LHb and its subnuclei (Quina et al., 2015). In order to directly compare the specific projections of the LHb GAD2 neurons to this established map, we injected CTB into the ventral raphe, NI and CGPn of mice in which all Gad2-expressing neurons have been genetically labeled with the fluorescent marker ZsGreen (*Gad2^Cre^* x *Ai6* mice; Materials and Methods; *N* = 13 mice of both sexes). Injection of CTB into the raphe, in an area including the DRI and caudal MnR (Fig. 8*A*) which receives dense LHb afferents (Figs. 5*J*,*K*, 6*F*), resulted in frequent labeling of LHb GAD2 neurons (Fig. 8*B–D*). Dual-labeled LHb neurons appeared throughout the rostrocaudal extent of the LHb, but were concentrated in the rostral to central part of the nucleus. Injection of CTB into the lateral CGPn (Fig. 8*E*), which receives dense LHb afferents (Figs. 4*T*,*U*, 5*M*, 6*K*,*L*), also labeled LHb GAD2 neurons (Fig. 8*F*,*G*). Neurons retrogradely labeled by injection of the right CGPn appeared in both hemispheres of the LHb, indicating that LHb neurons, including LHb GAD2 neurons, have both crossed and uncrossed projections, consistent with prior studies (Quina et al., 2015). Results were similar for CTB injection into the CGPn near the midline, incorporating the NI (Fig. 8*H–J*).

### Synaptic transmission by LHb GAD2 neurons

**Figure 9.**
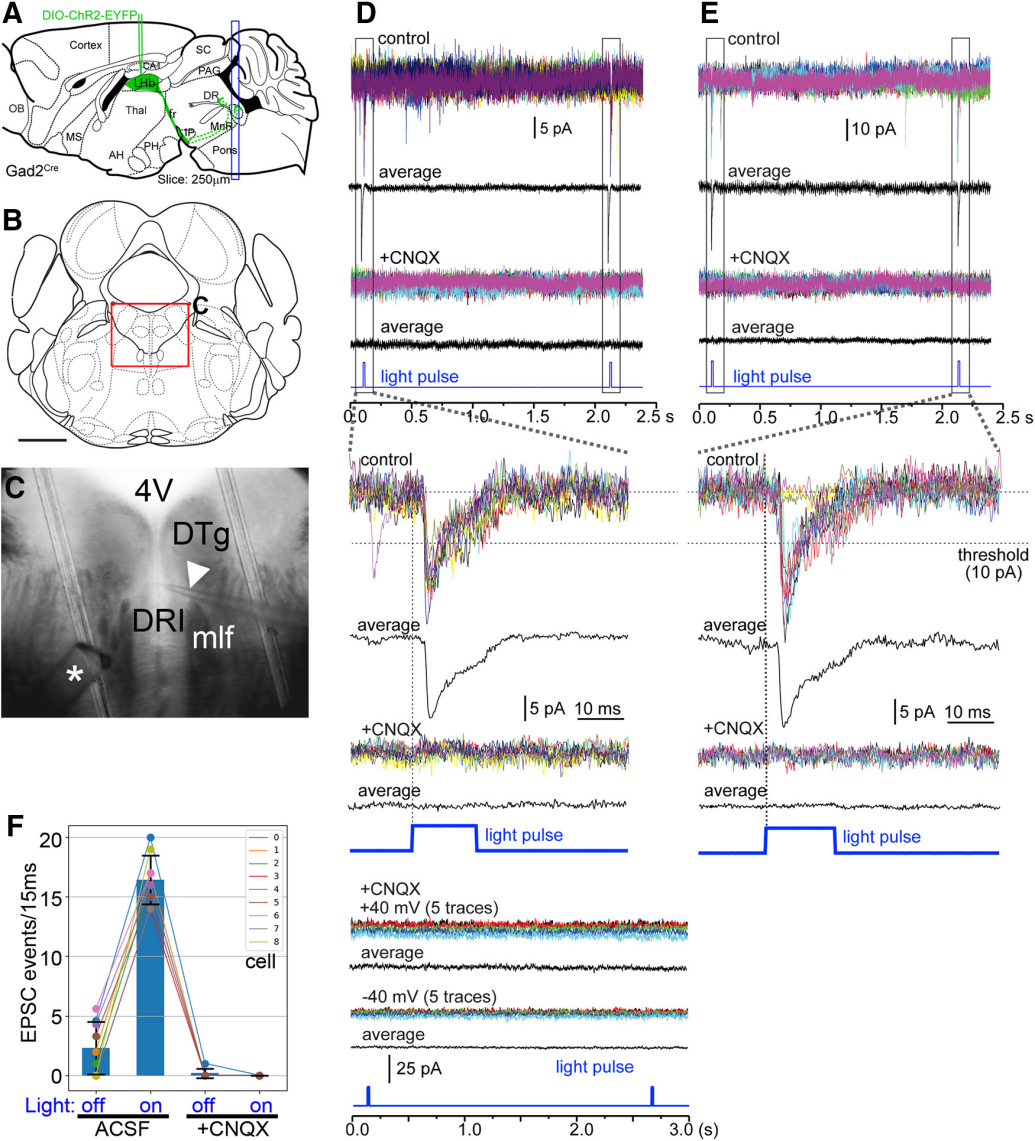
Excitatory synaptic transmission by LHb GAD2 neurons. Optogenetic stimulation of afferents to the mesopontine tegmentum was used to determine the neurotransmitter used for fast synaptic transmission by LHb GAD2 neurons. ***A***, Viral injection strategy for optogenetic recording. A Cre-dependent AAV encoding ChR2-EYFP was injected bilaterally into the LHb of *Gad2^Cre^* mice. The targeted coordinates were: AP -1.40, ML ±0.35, DV 2.65. Coronal tissue sections were prepared containing the caudal MnR, DRI, and CGPn, which contain the highest density of LHb GAD2 neuron afferents. ***B***, ***C***, Example of the location of a recorded cell in DRI. Asterisk in ***C*** indicates the tip of the optical fiber in the bath, and the arrowhead indicates the recording pipette. ***D***, upper panel, Voltage-clamp recording of a neuron from the area shown in ***C*** through 15 cycles of optogenetic stimulation (15/20 of the recorded cycles are shown) in ACSF and in ACSF with addition of the glutamate blocker CNQX (20 μm). Recordings in the upper panel were performed at a holding potential of –70 mV. In this case, 15/15 light pulses resulted in EPSCs. Addition of CNQX completely abolished the light response (lower traces). Lower panel, Voltage clamp recording of the same neuron at +40 and –40 mV relative to resting potential, in the presence of CNQX. No light-induced currents suggestive of fast GABAergic transmission were observed at either voltage. ***E***, Voltage clamp recording of another neuron in the tegmentum, in which 12/15 light pulses resulted in EPSCs which were abolished following bath application of CNQX (20 μm, lower traces). ***F***, Summary of optogenetic experiments: nine cells were recorded for 20 cycles of light stimulation in ACSF (left), and six of those cells were subsequently recorded for 20 cycles of light stimulation with CNQX in the recording bath (right). The *y*-axis shows the number of EPSC events (of amplitude >10 mA) occurring per 15 ms for 20 stimulus cycles under the stated conditions. 4V, fourth ventricle; DRI, dorsal raphe, inferior part; DTg, dorsal tegmental nucleus; mlf, medial longitudinal fasciculus.

Mouse LHb GAD2 neurons express GAD2, but show no evidence of VGAT expression; instead, they express the synaptic glutamate transporter VGluT2. Thus, we predicted that LHb GAD2 neurons would use only glutamate as a fast neurotransmitter. In order to assess synaptic transmission by LHb GAD2 neurons, we adopted an optogenetic strategy. The Cre-dependent AAV encoding a ChR2-EYFP fusion protein, EF1a.DIO.ChR2-EYFP, was injected into the LHb of *Gad2^Cre^* mice bilaterally (Fig. 9*A*), and coronal tissue slices containing the caudal MnR, inferior DR, and CGPn/NI, were harvested for electrophysiology. Under fluorescence optics, EYFP-labeled afferent LHb fibers could be visualized in all of these areas, and those areas of densest innervation were chosen for recording (Fig. 9*B*,*C*). Here, the goal was to determine the fast neurotransmitter used by the presynaptic LHb neurons, and the neurotransmitter identity of the recorded postsynaptic cells was not determined. Recorded neurons were held under voltage clamp conditions (Materials and Methods), and 20–30 cycles of optogenetic stimulation were applied, consisting of a 15-ms light pulse and a 2-s interstimulus interval (Fig. 9*D*,*E*). Some recorded cells responded to 50–100% of the light pulses (Fig. 9*D*), while others showed an EPSC following 100% of the optogenetic stimuli (Fig. 9*E*). Regardless of the response rate, recorded cells exhibited the same EPSC latency and amplitude in each cycle of stimulation, suggesting a repeated response to the same synaptic input. Of the nine recorded cells exhibiting EPSCs to >50% of light pulses, six cells were held in voltage clamp long enough to exchange the recording bath with ACSF containing the AMPA glutamate receptor inhibitor CNQX, which completely abolished the light evoked EPSCs in these cells (Fig. 9*D*,*E*, lower traces). Furthermore, in the presence of this glutamate blockade, no outward currents were observed that would suggest co-release of GABA (Fig. 9*D*,*E*). In the presence of CNQX, light-evoked GABA-mediated currents could in principle be obscured if the holding potential used to detect glutamate-mediated synaptic transmission lay near the equilibrium potential for chloride ions, and this is likely to be the case under the standard recording conditions used (−70 mV). However, light-evoked currents were also not observed in the presence of CNQX when more positive holding potentials of +40 or –40 mV were employed (Fig. 9*D*, lower panel), effectively ruling out fast GABA transmission. Statistical analysis showed a significant difference between the frequency of spontaneous EPSCs during the interstimulus interval and the frequency of EPSCs during the light stimulus [spontaneous frequency (mean ± SD) 2.3 ± 2.2 events/15 ms, light stimulus interval 16.4 ± 2.1 events/15 ms, expressed as the number of events per 20 stimulus cycles; *N* = 9 cells, paired *t* test: *t*_(8)_ = 26.01, *p* < 0.0001, *N* = 20 cycles], and also for the inhibition of light evoked EPSCs by CNQX (interstimulus interval 0.16 ± 0.4 events events/15 ms, stimulus mean, 0.0 events/15 ms; *N* = 6, two-way repeated measures ANOVA for six cells and four conditions: effect of light stimulation: *F*_(1,5)_ = 467.3, *p* < 0.0001, effect of CNQX: *F*_(1,5)_ = 202.5, *p* < 0.0001, and the interaction of these factors: *F*_(1,5)_ = 319.2, *p* < 0.0001) were highly significant (Fig. 9*F*). We conclude that, consistent with their gene expression profile, LHb GAD2 neurons use only glutamate as a fast synaptic neurotransmitter.

## Discussion

### Components of GABAergic transmission expressed in the LHb

Two surprising general observations emerge from the expression patterns of the three principal components of GABA synthesis and vesicular transport in the mouse and rat LHb. First, the expression patterns of GAD1 (Gad67), GAD2 (Gad65), and VGAT mRNA are quite distinct in mice and rats. Second, no LHb neurons in either species express any two of these GABAergic markers together. Instead, both the GAD2-expressing LHb neurons in the mouse, and the GAD1-expressing LHb neurons in the rat, express the vesicular glutamate transporter VGluT2.

Other than information published in databases, there is little prior work on GABAergic marker expression in the mouse LHb with which to compare this study. However, our findings in the Norway rat LHb are largely consistent with the data from prior studies, although not always with the interpretation of those data. Early studies of the rat LHb using GAD antisera (which did not distinguish GAD1 from GAD2) noted some immunoreactive LHb cell bodies, but focused mainly on the dense GABAergic afferents to this area (Gottesfeld et al., 1981; Belin et al., 1982). There have been several more recent references to the GABAergic neurons of the rat LHb, but no study has co-localized multiple GABAergic markers in LHb neurons, and the GABAergic nature of these cells has remained unclear (Zahm and Root, 2017). Our findings are entirely consistent with a prior *in situ* hybridization study that found GAD1-expressing neurons in the rat LHb, restricted to the lateral part of the nucleus, without GAD2-expression in any part of the nucleus (Brinschwitz et al., 2010). Although these were referred to as GABAergic neurons, VGAT expression was not examined in these neurons, and they were not further characterized. LHb GABAergic marker expression also has been shown to differ between rat species. A study comparing the expression of GAD2 in Norway rats with Nile grass rats, a diurnal rat species, confirmed the lack of GAD2 expression in the Norway rat, but showed expression of GAD2 mRNA in the latter species (Langel et al., 2018). GAD1 and VGAT expression were not examined in this study, and it was not determined whether grass rat GAD2 neurons co-express VGluT2.

Work from another laboratory, focused on vasopressin-expressing afferents to the LHb, has proposed a functional role for putative GABAergic neurons in the LHb (Zhang et al., 2016). *In situ* hybridization data for GAD1 in this study are consistent with the results reported here (Fig. 3*G*), with expression mainly in LHbL, and occasional GAD1-expressing cells in LHbM. Immunohistochemistry for GABA and GAD1 were also employed, but GAD2 and VGAT were not examined, so there is no evidence that these neurons can both produce and synaptically package GABA. In part of this work, conducted entirely in the rat, GAD2 expression in the mouse LHb, obtained from a database, was used to infer the location of rat GABAergic neurons in LHbM. Since GAD2 is not expressed in the rat LHb, this underscores the importance of understanding the species differences in the expression of GABAergic markers. These investigators have also described a population of VGAT (Slc32a1)-expressing neurons in the LHbM, which also express the estrogen receptor ERa and VGlutT2 (Zhang et al., 2018). These neurons are likely to coincide with the weakly VGAT-positive LHbM neurons shown in Figure 3*E*,*F*, which do not express GAD1 or GAD2. These neurons may have unique properties, but they are unlikely to be GABAergic in the usual sense.

Since it is hard to make a case that any class of rodent LHb neurons uses GABA for synaptic transmission, the physiological significance of GABA synthesis via GAD1 or GAD2 in the LHb remains unclear. Because mouse LHb GAD2 neurons also express VGluT2, we considered that these neurons might co-release GABA and glutamate, but in optogenetic whole-cell recordings from targets of LHb GAD2 neuron innervation, post-synaptic currents were entirely eliminated by AMPA receptor blockade. Co-release of GABA with other neurotransmitters is an emerging theme (Tritsch et al., 2016). GABA/glutamate co-release has been demonstrated for LHb-projecting neurons in the EP (Shabel et al., 2014; Wallace et al., 2017), and these neurons are also identified here in *Gad2^Cre^*/*Vglut2^FlpO^* tdT mice. However, these EP neurons are not comparable to LHb GAD2 neurons, in that drop-seq data show that they co-express VGluT2 together with GAD1, GAD2, and VGAT (Wallace et al., 2017), and thus have the appropriate synaptic transporters for both transmitters, which appear to be segregated in presynaptic areas (Root et al., 2018). GABA/glutamate co-release has also been detected in VTA neurons that project to the LHb (Root et al., 2014b), and these also clearly express both VGluT2 and VGAT for synaptic release of glutamate and GABA. Thus, these examples do not provide support for the synaptic release of GABA from neurons that express GABA synthetic enzymes without VGAT. Taken together, it appears unlikely that any population of neurons in the LHb of either rat or mouse are GABAergic in the conventional sense of using GABA as a synaptic neurotransmitter. The LHb does not appear to contain GABAergic interneurons resembling those in the cortex and hippocampus, or “GABAergic microcircuits” employing such cells (Zhang et al., 2016). The mouse LHb GAD2 neurons may more closely resemble a population of neurons in the LH that express the peptide melanin concentrating hormone (MCH), and also express GAD1 and VGluT2, but not VGAT (Mickelsen et al., 2017). Given the recent wide availability single-cell RNA sequencing, and of three-channel and four-channel FISH, and the application of these methods to generate brain-wide datasets, it will be interesting to see what other classes of neurons exhibit GAD1/2 expression without VGAT.

We also considered the possibility that LHb GAD-expressing neurons could release GABA via plasma membrane GABA transporters, which function to clear GABA from the intracellular space, but in principle are reversible (Scimemi, 2014; Savtchenko et al., 2015). The two plasma membrane GABA transporters found in the brain, GAT1 (Slc6a1) and GAT3/4 (Slc6a11), both show expression in the mouse LHb, where their transcripts are uniformly distributed rather than restricted to the areas expressing GAD2 (Allen Institute for Brain Science, 2012). This is consistent with the reported expression of GAT1 and GAT3 in astrocytes and afferent fiber terminals but not in resident neurons in the thalamus (De Biasi et al., 1998). Thus, it appears to be unlikely that GAT1 or GAT3 is available to transport GABA synthesized in LHb neurons to the extracellular environment.

### Marking LHb neurons that serve specific pathways

Regardless of whether a specific function can be identified for GABA synthesized in the LHb, GAD2 expression in the mouse, together with *Gad2^Cre^* transgenic mice, clearly provide a marker and an experimental system for manipulating a specific population of LHb neurons that projects predominantly to the raphe and pontine tegmentum, has relatively weak projections to the hypothalamus, and largely spares the VTA/RMTg. These results raise the question of whether the LHb GAD2 neurons in the mouse represent a significant pathway that is not present in the rat. This is impossible to assess definitively without an independent marker for these neurons that is expressed in both species. However, a prior study has shown that an injection of a conventional retrograde tracer into the rat caudal DR (DRC), which also labeled the adjacent pontine dorsal tegmentum (DTg, CGPn, and probably NI), marked cell bodies in the rat LHbM, in a pattern similar to that shown in Figure 8 (Sego et al., 2014). In contrast, retrograde tracing of LHb projections to the caudal VTA/RMTg has predominantly labeled LHbL, not LHbM (Kaufling et al., 2009; Bernard and Veh, 2012). Thus, these studies in the rat are consistent with the idea that the rat LHbM contains a population of raphe projecting/RMTg sparing neurons analogous to the mouse LHb GAD2 neurons, but which do not express GAD2.

Much of the interest in the LHb has been driven by its modulation of reward-related phenomena and DA activity via the GABAergic neurons of the RMTg (Jhou et al., 2009a; Kaufling et al., 2009; Balcita-Pedicino et al., 2011; Stamatakis and Stuber, 2012; Laurent et al., 2017; Li et al., 2019a,b). However, only a minority of LHb neurons, predominantly in LHbL, appear to map to the RMTg (Gonçalves et al., 2012; Quina et al., 2015). Thus recent attention has begun to focus more closely on the role of the LHb projections to its more extensive caudal targets, particularly the raphe 5HT pathway (Tchenio et al., 2016). In mice, tools based on LHb GAD2 expression should be able to provide new experimental insights into these pathways.

## Synthesis

Reviewing Editor: Christian Lüscher, University of Geneva Decisions are customarily a result of the Reviewing Editor and the peer reviewers coming together and discussing their recommendations until a consensus is reached. When revisions are invited, a fact-based synthesis statement explaining their decision and outlining what is needed to prepare a revision will be listed below. The following reviewer(s) agreed to reveal their identity: Marisela Morales.

The ms requires for the following points:

1. My main comment is that the authors did not conclusively show a lack of GABAergic influences in their electrophysiological recordings. They show that all post-synaptic currents (either inward or outward) are abolished after CNQX application. However, the patched cells were held at -70mV, possibly very close to both the potassium and chloride reversal potentials, given the pipette internal solution used. Hence, GABA-mediated currents could be difficult or impossible to see under these conditions. The EPSC amplitudes also appear quite small, leaving open the possibility (however unlikely given the lack of VGAT) that GABA currents were present that produced a shunting inhibition of the EPSC amplitude. Additional experiments optimized to reveal GABAergic currents would have been preferable, and in their absence, the authors would not be able to claim glutamate as the only fast transmitter, even though this seems likely based on the lack of VGAT.

2. The authors should discuss whether it is possible that GAD-expressing LHb neurons also make local collaterals, and whether, given the clearly unusual characteristics of these neurons, these might physiologically behave differently (or not) from the tegmental projections. Is it possible that GAD neurons somehow release GABA via some unknown mechanism that acts locally, but not distally?

3. The authors show in Fig. 5E that the tdT reporter is incompletely silenced in GAD2-Cre cells, leading to yellow labeling due to expression of both GFP and tdT. How do the authors know that the yellow label is due to incomplete silencing of tDT in GAD2-positive cells, rather than “leaky” recombination/activation of GFP in GAD2-negative cells? Admittedly, these possibilities might be hard to distinguish, but the possibilities should at least be noted. For example, if silencing is incomplete, then yellow labeling should decrease with extended survival after AAV injection (as there would be more time for Cre to excise the tdTomato gene). Conversely, if there is leaky recombination, then yellow labeling should increase over time. Do the authors have any evidence one way or the other?

4. The result section contains extent information corresponding to introduction, and previously published data currently in Fig. 1 (panels A-L) and Fig. 2 (panels A-D) need to be removed from the result section or reproduced with permission in a schematic fashion.
